# Hepatoprotective effect of *Pandanus odoratissimus* seed extracts on paracetamol-induced rats

**DOI:** 10.1080/13880209.2020.1865408

**Published:** 2021-01-06

**Authors:** Ernawati Sinaga, Ami Fitrayadi, Asrori Asrori, Sri Endarti Rahayu, Suprihatin Suprihatin, Vivitri Dewi Prasasty

**Affiliations:** a Faculty of Biology, Universitas Nasional, Jakarta, Indonesia; b Faculty of Biotechnology, Atma Jaya Catholic University of Indonesia, Jakarta, Indonesia

**Keywords:** Anti-hepatotoxicity, antioxidant, silymarin, histopathology

## Abstract

**Context:**

*Pandanus odoratissimus* Linn. (Pandanaceae) seed extract is known to have antioxidant activities. However, the potential hepatoprotective effect is still unclear.

**Objective:**

To investigate the hepatoprotection aspect of *P. odoratissimus* methanol extract towards paracetamol-induced rats.

**Materials and methods:**

Thirty male Sprague–Dawley rats were randomly divided into six equal groups: one group served as the healthy control and five groups with hepatotoxicity (hepatotoxic control and 4 treatment groups). The oral treatment of paracetamol-induced hepatotoxicity of 3 g/kg using three different concentrations of *P. odoratissimus* (300, 600 and 900 mg/kg), and silymarin (200 mg/kg) groups were administered once a day for 14 days. Enzyme activities and protein levels in serum were determined in rats at the end of the treatments. The histopathology of rat livers was observed under an electron microscope with 10× magnification.

**Results:**

*Pandanus odoratissimus* significantly decreased the serum glutamic-oxaloacetic transaminase (SGOT), serum glutamic pyruvic transaminase (SGPT), alkaline phosphatase (ALP) and γ-glutamyl transferase (GGT) activities in induced-paracetamol rat serum (*p* < 0.05). Moreover, *P. odoratissimus* significantly decreased total bilirubin and direct bilirubin levels (*p* < 0.05). It significantly blocked the decline of serum albumin and protein levels (*p* < 0.05). Histopathological changes amplified paracetamol-induced liver damage and the hepatoprotective effect of *P. odoratissimus* in the liver.

**Discussion and conclusions:**

*Pandanus odoratissimus* improved the hepatoprotective effect in a concentration-dependent manner by reducing related hepatic enzyme and protein markers, suggesting as a useful agent in hepatotoxicity treatment, and it can be generalized to a broader study population in different hepatotoxic animal models.

## Introduction

The liver is the centre of drug metabolism and it plays many critical functions such as cleansing toxins from the blood, producing immune agents to control infection, producing bile, and synthesizing proteins such as albumin and fibrinogen (Andreatos et al. 2017). It is well known that a substantial increase in steatosis and fibrosis usually leads to potentially lethal cirrhosis of the liver in humans (Turola et al. 2015). The high global prevalence of hepatopathy places it among the most severe diseases (Araújo et al. 2018). Although the pathogenesis of liver fibrosis is not entirely clear, there is no doubt that reactive oxygen species (ROS) play an essential role in pathological changes in the liver, particularly in cases of alcoholic and toxic liver diseases (Albano 2006; Contreras-Zentella and Hernández-Muñoz 2016). Biological membrane lipids are particularly prone to the ROS effect (Zadak et al. 2009). The peroxidation of unsaturated fatty acids in biological membranes leads to a decrease of membrane fluidity and to a disruption of membrane integrity and function, which is implicated in severe pathological changes (Erturk et al. 2014; Yan et al. 2015). Several endogenous protective mechanisms have been evolved to scavenge ROS and limit undesired cellular damage (Adams et al. 2013). However, since this protection may not be complete, or when the formation of ROS is excessive, additional protective mechanisms of dietary antioxidants may contribute to the health benefits (Ristow 2014). Therefore, many natural and artificial agents possessing antioxidative properties have been proposed to prevent and treat hepatopathies induced by oxidative stress (Lee et al. 2010; Kumarappan et al. 2011). There is increasing evidence for the hepatoprotective role of hydroxy- and polyhydroxy-organic substances, derived from fruits, vegetables, and some herbs (Bosek and Nakano 2003). Moreover, different kinds of tea leaves are the most popular non-alcoholic beverages that contain a wide range of various natural antioxidants (Shi et al. 2011; Busch et al. 2015).

Acute liver disease can be caused by the excessive use of paracetamol or acetaminophen (Larson 2007; Yan et al. 2018). Paracetamol is a drug that can act as antipyretic and give analgesic effects at a therapeutic dose (Badmann et al. 2012). However, excessive doses of paracetamol may cause liver damage (Naguib et al. 2014). Liver damage caused by paracetamol produced a toxic metabolite of *N*-acetyl-*p*-benzoquinone-imine (NAPQI) (Naguib et al. 2014). Paracetamol is metabolized through glucuronidation, sulphation, and oxidation reactions by cytochrome P-450 isozymes which primarily occur in the liver excreted through the kidney. The oxidation process by cytochrome P-450 isoenzymes will alter paracetamol into NAPQI (McGill and Jaeschke 2013). NAPQI will react with glutathione (GSH) in the liver cell (Soliman et al. 2014). However, excess doses may be toxic to the liver, and the damaged liver cells could be indicated by the elevated intracellular enzymes, especially aspartate aminotransferase (AST) and alanine aminotransferase (ALT) (Yoon et al. 2016). Acetaminophen poisoning effects such as nausea, vomiting, anorexia have toxic effects on the liver that can lead to death (Yoon et al. 2016). The increasing levels of ALT, AST, ALP, triglycerides, total bilirubin, as well as lower levels of protein and albumin are an early marker that more specific for detecting liver damage (Badrick and Turner 2016).

Bioactive constituents may serve as primary compounds for further development as hepatoprotective agents from toxic effects of paracetamol (Girish et al. 2009). Antioxidant compounds such as tannins, alkaloids, flavonoids, and polyphenols are known to improve liver function damaged by paracetamol (Abirami et al. 2015). The antioxidant compounds can reduce free radicals produced from the metabolism of paracetamol and prevent liver damage (Ramadan et al. 2013). Indonesia is widely available for the natural diversity of plants that can be used as raw material for herbal medicine (Antons and Antons-Sutanto 2009; Johan et al. 2017). *Pandanus* (Pandanaceae) can be found ranging from sandy beaches to the jungle plateau at an altitude of about 3500 m above sea level, and from secondary forests and meadows with shades of various soil from wet humus soil fertile, lime, peat bogs to the sandy soil where it is relatively dry and poor nutrient substances (Rachma et al. 2017). There are about 700 species in the genus *Pandanus*, 16 species are found on Java island (Callmander et al. 2012; Susanti et al. 2012). Four types of *Pandanus* were discovered in Ujung Kulon, Indonesia (Susanti et al. 2012). Species of *Pandanus odoratissimus* Linn. (Pandanaceae) is a type that has long been used by Indonesian people for a variety of purposes, ranging from spices to medicinal purposes (Raj et al. 2014; Gurmeet and Amrita 2015). *Pandanus* sp. have been reported to contain tannic compounds that have high antioxidant activities derived from the flavonoid compounds such as catechin and kaempferol, which can be used to fight free radicals in the body and prevent the pathological changes of liver disease (Okaiyeto et al. 2018). Thus, our study examined the hepatoprotective activity of *P. odoratissimus* in order to provide a scientific basis for the further usage of *P. odoratissimus* to have liver health benefits.

## Materials and methods

### Chemicals

Na-CMC, distilled water, methanol 98%, Dragendorff, reagent Mayer, iron (III) chloride, reagent Molish, lead (II) acetate, hydrochloric acid, Liebermann–Burchard, chloroform-isopronolol, amyl alcohol, magnesium and Whatman filter paper were analytical grade and provided from Merck. Paracetamol and silymarin were purchased from the local pharmacy. The kits for the assay of total bilirubin, direct bilirubin, albumin, and GGT were provided from Sigma Aldrich.

### Animals

Healthy male Sprague–Dawley rats (*n* = 30), 2–3 months old, bodyweight of approximately 200 g were provided from Cibinong Animal Facilities, Bogor, Indonesia and housed in suspended mesh-wired cages and fed with a standard rodent laboratory chow and water *ad libitum* under a 12 h light/dark cycle at room temperature. All experimental procedures were approved by the University Research Ethics Committee (2085/III/LPPM-PM.10.05/12/2019).

### Collection and identification of plant sample

*Pandanus odoratissimus* fruit sample (specimen code: S-Po-5) was collected from Calang, Aceh, and verified by a taxonomy expert, Dr. Sri Endarti Rahayu at Laboratory of Botany, Universitas Nasional in 2019. The other parts, such as fruit skin and flesh, were discarded, while seeds were collected and dried at room temperature. The dried *P. odoratissimus* seeds (POS) were soaked and mashed in a blender, to a sieve size of 60 mesh and dried. The powder was weighed, sealed in a plastic container and stored at room temperature.

### Preparation of plant extract

The seed powder of *P. odoratissimus* (1 kg) was macerated three times within 5 days with a total of 1500 mL of absolute methanol and stirred regularly. The supernatant was filtered with Whatman filter paper, collected, and concentrated with a rotary evaporator at 64 °C. The concentrated sample was stored in a sealed brown bottle at 4 °C for further analysis.

### Determination of total phenolic content

The total phenolic content was determined following previous studies with slight modifications (Baba and Malik 2015; Sembiring et al. 2017). The *P. odoratissimus* extract of 100 mg diluted in 10 mL distilled water to 10 mg/mL. About 1 mL of diluted *P. odoratissimus* extract was diluted into 1 mg/mL. *Pandanus odoratissimus* (0.2 mL) extract was added to 15.8 mL distilled water and 1 mL Folin–Ciocalteu reagent, then vortexed, incubated for 8 min and added with 3 mL of 10% Na_2_CO_3_ solution was settled for 2 h at room temperature. The UV-Vis absorbance was measured at *λ* = 765 nm.

### Determination of total flavonoid content

About 200 mg of *P. odoratissimus* seed (POS) extract was added with 1 mL of 0.5% (w/v) hexamethylenetetramine, 20 mL acetone, and 2 mL of 25% HCl, hydrolyzed by refluxing the extract within 30 min. The mixture was filtered and collected. The residue was re-refluxed with 20 mL acetone for 30 min, filtered, and collected. Acetone was added to reach volume at 100 mL. About 20 mL filtrate was transferred into separating funnel, added with 201 mL water, and extracted three times with each 15 mL ethyl acetate. Ethyl acetate fraction was collected and added with another 50 mL ethyl acetate in a volumetric flask. The reference solution of quercetin was prepared by taking 10 mL of stock solution, added with glacial acetic acid to reach volume at 25 mL. The sample solution was prepared by taking 10 mL of stock solution, added with 1 mL of AlCl_3_ and glacial acetic acid solution to reach volume at 25 mL (Baba and Malik 2015; Sembiring et al. 2017). Quercetin and sample measurements were done 30 min after the addition of AlCl_3_ using a spectrophotometer at *λ* = 425 nm. Total flavonoid content was expressed as mg quercetin equivalent (QE) per 100 g dried sample.

### Determination of antioxidant activity with DPPH

This assay is based on the ability of the sample to reduce DPPH (1,1-diphenyl-2-picrylhydrazyl) as the stable free radical agent. A total of 10 mg of sample was dissolved in 1 mL of DMSO (dimethyl sulfoxide) and vortexed. DPPH sample (100 mL) was loaded into a 96-well microplate, incubated at room temperature in the dark for 30 min. The control of 100 mL of ethanol solution was added in 100 mL of DPPH. Subsequently, the measurement was done in 517 nm at a time interval of 5 min, starting from 0 to 30 min using ELISA Reader. The power of antioxidants measured as the decreasing DPPH solution absorbance by the addition of the sample solution (Sembiring et al. 2017). DPPH solution absorbance value was calculated as the percentage inhibition by the following formula:

(1) $$\frac{\%\text{ Inhibition}=\text{Absorbance of standard}-\text{Absorbance of sample}\;\times \text{ 1}00\%}{\text{Absorbance of standard}}$$

### Experimental design

The *in vivo* experimental design of hepatoprotective power was performed using a complete randomized design (CRD), using animal models consisted of 30 Wistar male rats were divided into 6 groups as listed in Table 1.

**Table 1.** The research design of hepatoprotecting treatment using rat model cohort.

Rat cohort	Number of rats/cohort (*n*)	Induced by toxic agent (3 g/kg BW)	Treatment	Treatment dose (mg/kg BW)
Healthy control (KS)	5			
Hepatotoxic control (KP)	5	A single-dose paracetamol		
Silymarin (KSY)	5	A single-dose paracetamol	Silymarin	200
Sample treatment 1 (KE1)	5	A single-dose paracetamol	POS extract	300
Sample treatment 2 (KE2)	5	A single-dose paracetamol	POS extract	600
Sample treatment 3 (KE3)	5	A single-dose paracetamol	POS extract	900

### Preparation of Na-CMC suspension 0.5%

A total of 0.5 g of Na-CMC was added into 10 mL of hot distilled water and allowed to stand for 15 min to obtain a transparent feature, crushed to form a homogeneous gel, and diluted with distilled water. Distilled water was added to mark boundaries (Moghimipour et al. 2013). This suspension was used as a carrier of *P. odoratissimus* extract.

### Preparation of silymarin suspension

The silymarin powder was weighed 200 mg and added 0.5% Na-CMC gradually while crushed until it homogenous. The suspension was added with Na-CMC suspension of 0.5% (Ahmad et al. 2015).

### Preparation of paracetamol suspension

A total of 3 g of paracetamol powder was weighed and placed into a suspension in 0.5% Na-CMC in a mortar, crushed until homogeneity was achieved (Rishabha et al. 2010).

### Hepatotoxicity induction

The induction of hepatotoxicity was done by following previous methods (Freitag et al. 2015; Okokon et al. 2017) with minor modifications. Induction was performed using paracetamol orally, with a single dose of 3 g/kg BW. Paracetamol was suspended in 0.5% CMC. Paracetamol suspensions as much as 2 mL administered orally on day 12.

### Rat blood sampling

Rats were fasted for 12 h before blood sampling in day 15. The blood sampling procedures were following this stage: all treated rats were anaesthetized with ethyl ether in a sealed container in the palpability position of the rat heart, and blood was taken intracardially using a 3 mL syringe. Blood samples were centrifuged for 10 min at 4000–5000 rpm to obtain the serum. The serum was used to determine the levels of total protein, total bilirubin, direct bilirubin, albumin, and enzyme activities of GGT, SGOT, SGPT, ALP.

### Determination of total bilirubin, direct bilirubin, albumin, total protein content, GGT, SGOT, SGPT and ALP in rat serum

Determination of total bilirubin, direct bilirubin, albumin, total protein content, GGT, SGOT, SGPT, and ALP in rat serum was done by following the previous study (AlSaid et al. 2015). The working methods and procedures outlined as follows:

### Determination of total bilirubin level

About 25 μL of serum was added into 1000 μL of reagent 1 containing phosphate buffer and NaCl, incubated for 5 min at 37 °C. The reagent 1 was added by 250 μL of reagent 2 containing 2,4-chlorophenyl diazonium and HCl, incubated for 5 min, and homogenized. The absorbance was measured at 546 nm, and total bilirubin was calculated.

### Determination of direct bilirubin levels

About 50 μL of serum was added in 1000 μL of reagent 1 direct bilirubin (EDTA-Na_2_ and sulphamic acid), incubated for 3–5 min at 37 °C and combined with 250 μL reagent 2 direct bilirubin (2.4-chlorophenyl diazonium, HCl and EDTA-Na_2_) until homogenized. The absorbance was measured at 546 nm.

### Determination of albumin concentration

About 10 μL of serum was added in 1000 μL of reagent Albumin (buffer citrate, pH 4.2, and bromocresol green), incubated for 10 min at 37 °C, and measured at 546 nm.

### Determination of total protein content

About 20 μL of serum was added in 1000 μL of reagent 1 of total protein (sodium hydroxide, potassium sodium tartrate), incubated for 5 min at 37 °C, added with 250 μL reagent 2 of total protein (sodium hydroxide, potassium sodium tartrate, potassium iodide, Cu-sulphate) and homogenized. The absorbance was measured at 540–546 nm. The measurements of SGOT, SGPT, ALP, and total protein levels were conducted in Laboratory Presidential RS Gatot Subroto Army Hospital in Central Jakarta (Biotechnica 3500, International Federation of Clinical Chemistry [IFCC]).

### Determination of GGT activity

About 100 µL serum was added with 1000 µL reagent 1 (glycilglycine and tris, pH 8.25), incubate for 1 min at 37 °C, then add 250 µL reagent 2 (l-glutamic acid γ-(3-carboxy-4-nitroanilide) then homogenized. The absorbance was measured at 546 nm.

### Determination of SGOT activity

About 100 µL of serum was added in 1000 µL of reagent 1 SGOT (Tris, l-aspartate, MDH, LDH), incubated for 5 min at 37 °C, added with 250 µL reagent 2 of AST (2-oxoglutarate, NADH), then homogenized. The absorbance was measured at 365 nm.

### Determination of SGPT activity

About 100 μL of serum was added in 1000 μL of reagent 1 SGPT (Tris, l-alanine, LDH), incubated for 5 min at 37 °C, added with 250 μL reagent 2 SGPT (2-oxoglutarate, NADH) and homogenized. The absorbance was measured at 365 nm.

### Determination of ALP activity

Serum (20 μL) was added into 1000 μL of reagent 1 ALP (2-amino-2-methyl-1-propanol, magnesium acetate, zinc sulphate, HEDTA), incubated for 5 min at 37 °C, added with 250 μL reagent 2 of ALP reagent (*p*-nitrophenylphosphate) and homogenized. The absorbance was measured at 400–420 nm.

### Histological analysis

Histological analysis was done at the end of the treatment period. Both control and experimental rats were euthanized using carbon dioxide asphyxiation. The livers were collected from all groups, fixed in saline with 10% formalin, dehydrated in ethyl alcohol, rinsed in xylol, and mounted in molten paraplast at 58–62 °C. Five-micron sections were collected, stained with haematoxylin & eosin (H&E), and evaluated for any structural changes under a bright-field microscope (El-Sayyad et al. 2009) with 10**×** magnification.

### Statistical analysis

The statistical analyses were conducted with GraphPad Prism 7. The one-way analysis of variance (ANOVA) followed by the Tukey test was used to determine the level of significance (*p <* 0.05) with the 95% confidence interval, where all data was expressed as mean ± standard deviation (SD).

## Results

### Determination of total flavonoids, total phenolics and antioxidant power of *P. odoratissimus* extract

The quantitative results of total flavonoids, and total phenolics, of *P. odoratissimus* extract, were 0.5 and 11.62%, respectively (Table 2). The results of the examination of the antioxidant power of the extract of *P. odoratissimus* showed IC_50_ values <31.25 µg/mL which was weaker than quercetin standard (3.52 µg/mL).

**Table 2.** Phytochemical substituent value of *P. odoratissimus* extract.

Constituent	IC_50_ (ppm)	Percentage (w/w)
Total flavonoids		0.5%
Total phenolics		11.62%
Antioxidant power	31.25	

### Hepatoprotective effect of *P. odoratissimus* extract

The statistical measurements of SGOT, GPT, ALP, and GGT levels in rat serum showed that the healthy and hepatotoxic control groups were significantly different (*p <* 0.05), as listed in Tables 3 and 4. The results showed that intoxication by giving 3 g/kg BW of paracetamol has succeeded in damaging or intoxicating the liver. An increase of SGOT, SGPT, ALP and GGT activity in hepatotoxic-induced rat serum has confirmed the occurrence of liver damage due to paracetamol intoxication.

**Table 3.** The effect of *P. odoratissimus* seed (POS) extracts towards SGOT and SGPT activities.

Cohort	Treatment	SGOT (U/L)	SGPT (U/L)
KS	Healthy control	161.40 ± 4.72^a^	62.00 ± 5.20^a^
KP	APAP 3 g/kg	897.25 ± 47.36^b^	1636.20 ± 50.13^b^
KSY	APAP + Silymarin	479.00 ± 7.78^c^	334.40 ± 19.45^c^
KE1	APAP + POS 300 mg/kg	476.75 ± 36.87^c^	453.20 ± 22.12^d^
KE2	APAP + POS 600 mg/kg	190.80 ± 14.26^a^	156.40 ± 19.02^c^
KE3	APAP + POS 900 mg/kg	109.80 ± 10.70^a^	49.20 ± 2.86^a^

Data are presented as mean ± SD (*n* = 5). Different superscript letters indicate significant difference (*p* < 0.05) between each other. The same superscript letters show no significantly different (*p* > 0.05) between each other.

**Table 4.** The effect of *P. odoratissimus* seed (POS) extracts towards ALP and GGT activities.

Cohort	Treatment	ALP (U/L)	GGT (U/L)
KS	Healthy control	318.20 ± 54.86^a^	3.00 ± 1.000^a^
KP	APAP 3 g/kg	672.00 ± 18.88^b^	11.80 ± 2.049^b^
KSY	APAP + Silymarin	569.80 ± 9.34^c^	7.80 ± 0.837^c^
KE1	APAP + POS 300 mg/kg	436.80 ± 8.56^c^	6.80 ± 1.095^c^
KE2	APAP + POS 600 mg/kg	372.80 ± 12.60^a^	4.80 ± 1.095^a^
KE3	APAP + POS 900 mg/kg	311.80 ± 20.75^a^	2.80 ± 0.837^a^

Data are presented as mean ± SD (*n* = 5). Different superscript letters indicate significant difference (*p* < 0.05) between each other. The same superscript letters show no significantly different (*p* > 0.05) between each other.

**Figure 1.**
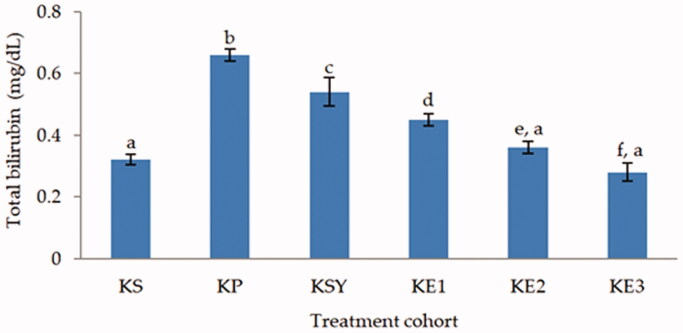
Effect of *P. odoratissimus* seed (POS) extract on total bilirubin of serum rats induced by paracetamol. KS: healthy control; KP: hepatotoxic control; KSY: silymarin control; KE1: POS extract 300 mg/kg; KE2: POS extract 600 mg/kg; KE3: POS extract 900 mg/kg.

The SGOT and SGPT activities in the serum of rat intoxication with paracetamol were much higher than in the healthy rat. Statistical results showed that SGOT and SGPT activities in the healthy and hepatotoxic control groups were significantly different. Figure 1 also shows that the administration of *P. odoratissimus* extract can inhibit the increase of SGOT and SGPT activities caused by paracetamol intoxication. The one-way ANOVA analysis revealed that there was a significant effect (*p* < 0.05) between the SGOT and SGPT activities of treated groups compared to the hepatotoxic control group. The average of SGOT activity in the hepatotoxic control group was 885.20 U/L while in the groups treated with plant extracts of 300, 600 and 900 mg/kg BW were 463.60 U/L, 190.80 U/L and 109.80 U/L, respectively. Moreover, the average of SGPT activity in the hepatotoxic control group was 1636 U/L while in the groups treated with plant extracts of 300, 600 and 900 mg/kg BW were 334 U/L, 156 U/L, and 49 U/L, respectively (Table 3).

The administration of *P. odoratissimus* extracts with doses of 300, 600 and 900 mg/kg and silymarin 200 mg/kg BW significantly decreased the ALP and GGT activities in paracetamol intoxication. It appeared that the ALP and GGT activities treated with *P. odoratissimus* extract and silymarin were significantly different (*p* < 0.05) compared to the hepatotoxic group. The ALP and GGT activities of rat models that were given 600 and 900 mg/kg BW extracts were not significantly different from the healthy rats (Table 4).

**Figure 2.**
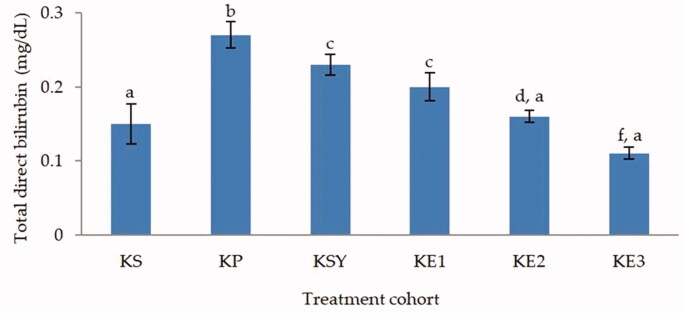
Effect of *P. odoratissimus* seed (POS) extract on total direct bilirubin of serum rats induced by paracetamol. KS: healthy control; KP: hepatotoxic control; KSY: silymarin control; KE1: POS extract 300 mg/kg; KE2: POS extract 600 mg/kg; KE3: POS extract 900 mg/kg.

The total bilirubin and direct bilirubin levels (*p* < 0.05) showed a significantly increase in the toxic rat-induced paracetamol group, as shown in Figure 1 and Figure 2. The administration of *P. odoratissimus* extract with a dose of 300, 600 and 900 mg/kg and silymarin 200 mg/kg can inhibit the increase of paracetamol intoxication. The levels of total bilirubin and direct bilirubin in the rat groups treated with extract and silymarin were significantly different (*p* < 0.05) from the hepatotoxic rat group. Moreover, the total bilirubin and direct bilirubin levels of rat groups treated with *P. odoratissimus* extract in doses of 600 and 900 mg/kg were not significantly different compared to the healthy rat group (*p* > 0.05).

**Figure 3.**
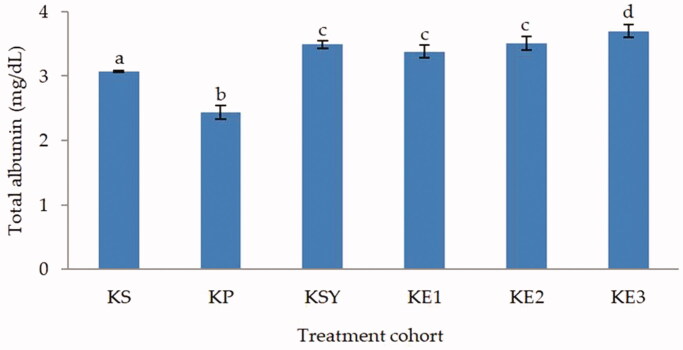
Effect of *P. odoratissimus* seed (POS) extract on total albumin levels of serum rats induced by paracetamol. KS: healthy control; KP: hepatotoxic control; KSY: silymarin control; KE1: POS extract 300 mg/kg; KE2: POS extract 600 mg/kg; KE3: POS extract 900 mg/kg.

**Figure 4.**
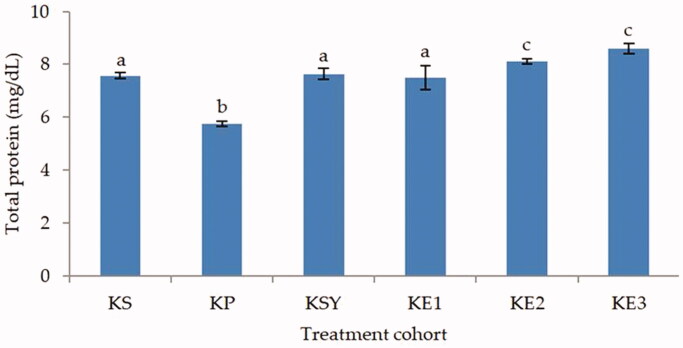
Effect of *P. odoratissimus* seed (POS) extracts on total protein levels of serum rats induced by paracetamol. KS: healthy control; KP: hepatotoxic control; KSY: silymarin control; KE1: POS extract 300 mg/kg; KE2: POS extract 600 mg/kg; KE3: POS extract 900 mg/kg.

The total albumin and total protein levels (*p* < 0.05) showed significantly different in the toxic rat-induced paracetamol group compared to healthy, silymarin, and *P. odoratissimus* treated rat groups as shown in Figures 3 and 4. The administration of *P. odoratissimus* extract with a dose of 300, 600 and 900 mg/kg and silymarin 200 mg/kg can inhibit the increase of paracetamol intoxication. The levels of total albumin and total protein in the rat groups treated with extract and silymarin were significantly different (*p* < 0.05) from the hepatotoxic rat group. Moreover, *P. odoratissimus* extract with doses of 300, 600 and 900 mg/kg was not significantly different compared to the healthy rat group (*p* > 0.05). Thus, it can be concluded that the administration of *P. odoratissimus* extracts in all doses can significantly recover total albumin and total protein levels in rat serum to inhibit the effects of paracetamol intoxication.

### Histopathology of rat liver

**Figure 5.**
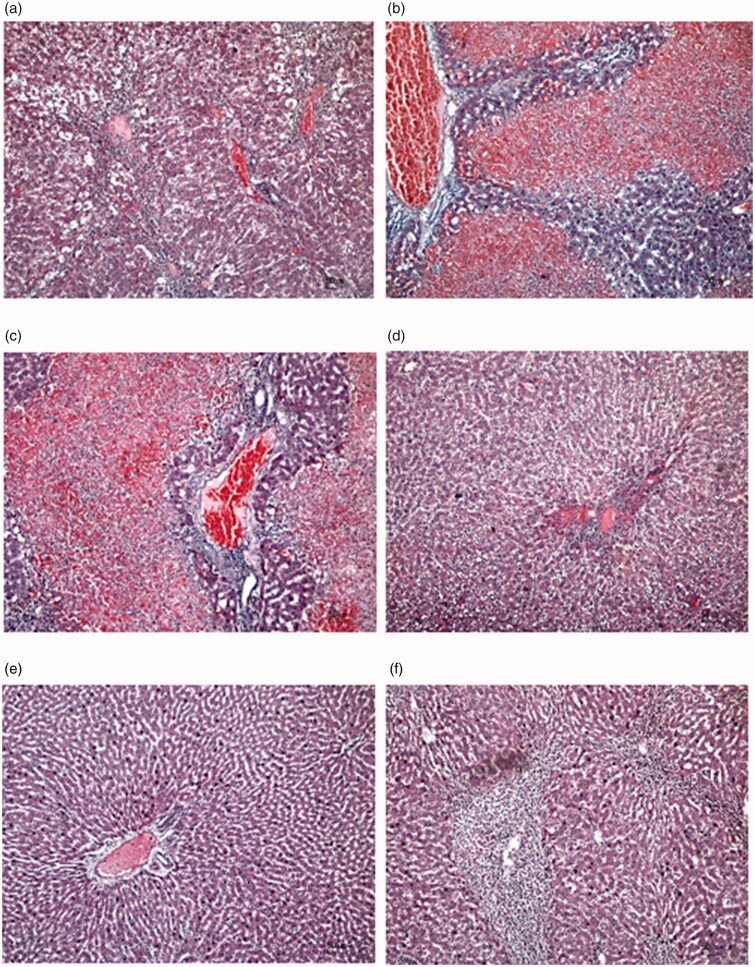
Histological profiles of liver cells in 6 rat cohorts with 10× magnification in focus assay: (a) healthy control; (b) hepatotoxic control showed massive inflammation liver cells; (c) silymarin control at 200 mg/kg indicated inflammation liver cells; (d) *P. odoratissimus* extract of 300 mg/kg; (e) *P. odoratissimus* extract of 600 mg/kg; (f) *P. odoratissimus* extract of 900 mg/kg. Subfigures (d)–(f) demonstrated the healing of liver cells after the extract treatments towards paracetamol intoxication.

The histopathology of rat livers from 6 groups was done by observation under an electron microscope with 10x magnification. It can be seen that rat liver cells in healthy control were having a similar appearance with *P. odoratissimus* extracts of 3,00,600 and 900 mg/kg, respectively. Meanwhile, rat liver cells in hepatotoxic control and silymarin control showed similar patterns that dominated with red colours in the cells, indicating damaged cells caused by paracetamol intoxication (Figure 5). The healthy control group shows normal hepatic cells, while paracetamol administrations indicate inflammation and gross necrosis. Moreover, treatment with *P. odoratissimus* extracts reversed the hepatic lesions caused by paracetamol intoxication to a large extent.

## Discussion

Flavonoids belong to phenolic compounds commonly found in several parts of plants, mostly in plant cells that require photosynthesis. Flavonoids are known to have biological activities such as antiviral, antibacterial, anticancer, antioxidant, anti-inflammatory, and hepatoprotector (Tapas et al. 2008; Zhong et al. 2011). Total flavonoids and phenolic compounds in this study were higher (0.5 and 11.62%, respectively) compared to the previous study (Adkar and Bhaskar 2014). Phenolic compounds in general can reduce free radicals and have a variety of functional properties such as anti-inflammatory, hypo-allergenic, antithrombotic, hepatoprotective, and anticarcinogenic properties (Itoh et al. 2009; Plundrich et al. 2015; Helkar et al. 2016). Phytochemical constituents of *P. odoratissimus* methanol extract identified by column chromatography were phenolic derivatives (vanillin, 2 (*E*)-3-(3′-methoxy-4-hydroxyphenyl)-prop-2-enal, 4-hydroxy-3-(2′,3′-dihydroxy-3′-methyl-butyl)-benzoic acid methyl ester), benzofuran derivative (3-hydroxy-2-isopropenyl-dihydrobenzofuran-5-carboxylic acid methyl ester), and lignans (eudesmin, kobusin, pinoresinol, epipinoresinol, de-4′-*O*-methyleudesmin, and 3,4-bis(4-hydroxy-3-methoxy-benzyl)-tetrahydrofuran). These phytoconstituents have bioactivities as anticancer and antioxidant (Adkar and Bhaskar 2014).

The antioxidant power of the *P. odoratissimus* methanol extract in this study was also higher (IC_50_ = 31.25 ppm) than reported in the previous study where IC_50_ of *P. odoratissimus* ethanol extract was 36.70 ppm (Penu et al. 2020). Antioxidant activity expressed by IC_50_ is a value of the extract concentration that can capture 50% of free radicals. The smaller amount of IC_50_ indicates the higher antioxidant activity, thus effective as a free radical scavenger. Bioactive compounds become powerful antioxidant agents if they have IC_50_ value less than 50 µg/mL, strong if IC_50_ is 50–100 µg/mL, moderate if IC_50_ is 100–150 µg/mL, and weak if IC_50_ value within 151–200 µg/mL (Setha et al. 2013). Therefore, *P. odoratissimus* extract was categorized as a powerful antioxidant agent (31.25 µg/mL), but weaker than quercetin control (3.52 µg/mL).

The hepatoprotective activity of *P. odoratissimus* extract was assessed based on the activity of SGOT, SGPT, ALP, and GGT in rat serum, which was induced by paracetamol. Paracetamol is one of the compounds that are hepatotoxic at a high dose. It is also well-known that paracetamol is a drug with a proportion of 39–57%, causing acute liver failure in the United States and the United Kingdom (Tittarelli et al. 2017).

About 25% of paracetamol is bound to plasma proteins and partly metabolized by liver microsome enzymes. Paracetamol can be conjugated with glucuronic acid in the liver. At therapeutic doses, 5–15% of paracetamol will be converted by cytochrome P450 to a highly reactive metabolite, *N*-acetyl-*p*-benzoquinonymin (NAPQI). NAPQI will be quickly detoxified by conjugation with cell glutathione (Ohba et al. 2016). This reaction results in the formation of cysteine and mercapturic acid excreted in the urine. At the time of paracetamol intoxication, the amount and speed of NAPQI formation exceed the ability of the liver to replenish glutathione reserves. Glutathione depletion causes NAPQI to bind covalently to the cysteine group in proteins. The main target is the mitochondrial protein, which results in damage to ATP production. Mitochondrial dysfunction will also produce reactive oxygen species (ROS) and reactive nitrogen species (RNS), which results in oxidative stresses. These oxidative stresses cause hepatotoxicity (Moyer et al. 2011; Yoon et al. 2016; Tsai et al. 2018).

SGOT, SGPT, ALP, and GGT are enzymes that normally present in liver cells, and only in a small amount in the blood. But if the liver cell membrane is damaged, these enzymes will come out of the liver cells and penetrate into the blood. It will increase the enzyme activities in the bloodstream and could be markers of breaking liver cell membranes (Washington and Van Hoosier 2012).

Serum glutamate oxaloacetate transaminase (SGOT) is a liver enzyme that helps produce protein. This enzyme catalyzes the transfer of an amino group from aspartate to α-ketoglutarate to produce oxaloacetate and glutamate. In addition, this enzyme is also found in other organs such as the heart, skeletal muscle, brain, and kidney. The damage to one of these organs can cause an increase in the enzymes in the blood. Normal SGOT levels are in the range of 7–40 U/L. This enzyme also helps in detecting liver cell necrosis. However, it is considered to be a less specific marker for liver cell damage due to this enzyme can also describe abnormalities in the heart, skeletal muscles, brain, and kidneys (Lin et al. 2011).

Serum glutamate pyruvate transaminase (SGPT) is the most commonly used marker of liver toxicity. SGPT is a liver enzyme that plays an essential role in amino acid metabolism and gluconeogenesis. This enzyme catalyzes the transfer of an amino group from alanine to α-ketoglutarate to produce glutamate and pyruvate. Normal SGPT levels are in the range of 5–10 U/L. The increased SGPT level occurs in liver damage. Measurement of SGPT levels is a more specific test to detect liver abnormalities due to it is mainly found in the liver. This enzyme is also found in skeletal and heart muscle, but its activity is lower. Thus, SGPT can detect liver cell necrosis (Lin et al. 2011).

The treated groups with plant extracts in 3 doses were significantly different with hepatotoxic group in decreasing SGOT and SGPT activities. Meanwhile, the administration of *P. odoratissimus* extracts with doses of 600 and 900 mg/kg BW was not significantly different with the healthy control group in decreasing SGOT activities in serum but only a dose of 900 mg/kg BW was not significantly different with the healthy control group in decreasing SGPT activity.

Alkaline phosphatase (ALP) is an enzyme produced mainly by the liver epithelium and osteoblasts (new bone-forming cells), this enzyme also comes from the intestine, proximal renal tubules, specifically in pregnant women, ALP is produced from the placenta. ALP is secreted through the bile duct. ALP may increase in blood serum when there is an obstacle in the bile duct (cholestasis). An abnormally elevated ALP level indicates liver or bone disease (Corathers 2006). If there is mild damage to the liver cells, ALP levels may rise, but it increases in acute liver disease.

**γ**-Glutamyl transferase (GGT) is an enzyme that is bound to cell membranes that catalyze the reaction of γ-glutamyl transfer from a peptide to amino acids or other peptides. GGT is not only found in the membranes of liver cells but also found in membranes of various other tissues, including the membranes of proximal tubular cells of the kidney, pancreas, intestine, and spleen. GGT is mainly located in the biliary epithelial cells and the apical membrane of hepatocytes in the liver. However, GGT in serum is produced from the liver. Therefore, the increasing GGT activity in the serum is most likely derived from the liver.

The administration of *P. odoratissimus* extracts with doses of 600 and 900 mg/kg BW could inhibit the toxic effects of paracetamol. The administration of *P. odoratissimus* extract with a dose of 300 mg/kg BW has a hepatoprotective power of approximately equivalent to silymarin 200 mg/kg BW, where ALP and GGT activities in the serum were not significantly different. The ALP and GGT activities of rat models that were given 600 and 900 mg/kg BW extracts were not significantly different from the healthy rats that possessed the inhibition of paracetamol intoxication (Table 4).

Total bilirubin is the total amount of bilirubin in the blood, including conjugated and unconjugated bilirubin. Conjugated bilirubin is also called direct bilirubin. Direct bilirubin is soluble in water and can be found in urine. Indirect bilirubin is also called unconjugated bilirubin. Indirect bilirubin is not soluble in water. Indirect bilirubin is not easily dissolved in water. Thus, it is not found in urine (Washington and Van Hoosier 2012; Valášková and Muchová 2016).

Albumin is the most abundant protein in plasma, consisting of approximately 50% of the total plasma protein. Albumin is a single polypeptide with about 585 amino acids. Its molecular weight is around 66 kDa. Clinically, reduced albumin in blood plasma can affect redox balance, haemostatic disorders, impaired liver metabolism, acid-base imbalances, and, especially, reduce detoxification capacity and antioxidant activity (Bernardi et al. 2014). The total bilirubin and direct bilirubin levels of rat groups treated with *P. odoratissimus* extract in doses of 600 and 900 mg/kg were not significantly different compared to the healthy rat group (*p* > 0.05). Thus, the administration of *P. odoratissimus* extracts with doses of 600 and 900 mg/kg are potential as hepatoprotective.

Total protein concentration and haematocrit values increase in cases of dehydration, followed by an increase in albumin and globulin concentrations (Jackson et al. 2013). Decreased total protein concentration can also be caused by malnutrition and mal-absorption, liver disease, chronic and acute diarrhoea, burning, hormonal imbalance, kidney disease (proteinuria), low albumin concentration, and low globulin concentration (Meiklejohn et al. 2019). An increase in total bilirubin levels and direct bilirubin activity and a decrease in albumin levels and total protein level in the hepatotoxic-induced rat is indicating damage to the integrity or function of liver cells due to paracetamol intoxication (Valášková and Muchová 2016; Yoon et al. 2016).

Histopathology findings revealed widespread necrosis was reduced to few inflammatory liver cells in the rats treated with *P. odoratissimus* extracts. Besides the seed part, roots part of the plant also possessed significant protection against paracetamol-induced hepatotoxicity in rats at 400 mg/kg (Mishra et al. 2015). The histopathological study showed a decrease in the degree of necrosis in the rats treated with *P. odoratissimus* extracts. This is due to *P. odoratissimus* contains hepatoprotective agents such as flavonoids, coumarin, tocopherol, and polyfunctional organic acids (Chilakwad et al. 2008; Itoh et al. 2009; Londonkar and Kamble 2009; Plundrich et al. 2015; Helkar et al. 2016).

## Conclusions

The *P. odoratissimus* extract contains 0.50% (w/w) of total flavonoid, 11.62% (w/w) of total phenolics and antioxidant power with IC_50_ value < 31.25 ppm. Moreover, *P. odoratissimus* extract has a hepatoprotective effect that could inhibit the increase of SGOT, SGPT, ALP and GGT enzyme activities also could inhibit the increase of total bilirubin and direct bilirubin levels in rat serum. In contrast, it showed a decrease of total albumin and total protein levels in rat serum, which indicated the recovery of paracetamol intoxication. Histopathological changes led to liver damage caused by paracetamol and treatment of *P. odoratissimus* could amplify hepatoprotective effects in the liver.
